# Extra Supplement.—The Nursing Mirror

**Published:** 1888-12-22

**Authors:** 


The Nursing Mirror.

All Contributions for this Supplement should be addressed "The Nursing Mirror," care of The Editor, 38, Old Jewry, E.C.

### En Passant.

CONCERT.—In the beginning of the month, a delightful concert was given at the Kensington Town Hall by the friendly societies, in aid of the Kensington District Nursing Association. Mr. Frank Braine was the conductor, and the Hon. and Rev. E. Carr Glyn occupied a prominent position. This nursing association is doing a great deal of good, but is crippled by want of funds.

DECORATIONS.—It is very trying to add the labour of twining and putting up evergreens to the usual ward work of a nurse, and yet the ward where a nurse does not herself help with the decoration is never well nor suitably done. We all have to take up an added burden of work as Christmas draws near, and remembering the season, the least we can do is to be cheerful under the extra trouble. It is wonderful how the merry heart will work on, while "the sad tires in a mile-a'"; and the holly put up by a cross nurse will scarcely look cheerful to the poor patients. Outside help is very valuable in hospitals at this time of the year; but yet let every nurse do her best towards making Christmas a festive season, and she will find she has gained a step in inward grace.

NURSES AND ADVERTISEMENTS.—The ways of the vendor of patent medicines are past finding out, still we did not expect them to get hold of nurses to puff their advertisements. Just now there appears in many country papers a paragraph purporting to be quoted from some other paper, and headed "A Nurse's Report." Then follow two stories, one of a nurse of "Swaffham" (which name calls up recollections of Dickens), and then a quotation presumably from a letter written by Sister Caroline, St Andrew's Hospital, Clewer. Now, if there is anyone who calls herself Sister Caroline at that address, we hope she now sees her error, and will no longer lend her name to such unprofessional usage. This placarding of the opinions of so-called nurses, on the merits of patent medicines, is on the increase, and all those of our readers who have the interest and dignity of their calling at heart, should do their best to discourage it.

CONVERSAZIONES.—There is one very difficult point about conversaziones in hospital life which those who promote them do not seem to consider; and this is the relative position of doctors and nurses. So long as a nurse is in uniform, she therefore, as it were, on business, takes a lower standard than a doctor; so soon as a nurse is out of uniform, and in her character of a private lady, she takes position by right of her sex. Now, when nurses in uniform are brought socially into contact with the doctors for whom they work, there is awkwardness on both sides. You see the doctors delivering a few kindly words, you see the nurses lacking courage to touch on conversational topics; both feel they are placed in a false position. As a rule the visiting medical staff of a hospital does not care to be invited to meet the nurses in a professional capacity, and though the house staff may look forward to making better acquaintance with women whose work they admire, these occasions are seldom fraught with hearty pleasure to either. Scandal about nurses and students is so common amongst the Society papers, who are always marrying them off in pairs, that caution to avoid real cause of complaint, cannot be too carefully sought. The nursing staff, fully represented, and the medical staff, as at the B. N. A. on the 6th inst., conspicuous by the absence of a majority of its members, is a comm[illegible]n sight at hospital conversaziones.

SUICIDE OF A NURSE.—An inquest was held in London on Monday last on the body of Mary Creed, at one time a hospital nurse. The deceased went abroad six years ago and contracted African fever, from which she suffered at intervals after her return to England. Her last post was that of matron to an orphan asylum near Shelburn. The manner of suicide was extraordinary, for the unhappy woman had tied a piece of tape to the bed-post, and then wound the tape tightly round her neck and hung her head over the side of the bed.

HURRAH FOR THE PENSION FUND!—This fund is still progressing rapidly. At a meeting of the council held yesterday, Mr. Walter H. Burns in the chair, the honorary manager reported that the number of applications for pensions and sick pay up to date was 574, of whom 418 had paid their contributions, amounting to £6,145, of which £2,018 had been received during the month of November. Sixty-five applications had been received since the last meeting of the council, and 72 were accepted at the present meeting. It was reported that the funds invested amounted to upwards of £30,000.

DISTRICT NURSES.—The Epsom committee of those interested in district nursing has just held its first annual meeting, to congratulate itself on the success of its work. The district nurse at Epsom has done much good during the last year, and fulfilled the expectations of those who engaged her. It only costs £105 a-year to keep a parish nurse, so all small towns which have no hospital might indulge in this little luxury for the poor. From all sides we hear of district nurses being wanted, and of grants being wanted from the jubilee fund towards starting district nursing. Soon there will be no corner of England to which the trained nurse has not penetrated. Those who seek help from the jubilee fund must wait awhile; that scheme is not yet in working order.

THE BRITISH NURSES' ASSOCIATION.—We have received considerable correspondence on the subject of our last week's notice of the Grosvenor Gallery Conversazione, and herewith beg to intimate to our indignant readers that because our correspondents enjoyed the conversazione, and said so, we have yet remained faithful to the trust they evidently repose in us. We do not admire certain aims and objects of the Association any more now than before, and are still as much opposed to its plan of registration as all far-seeing people must be. But there is no necessity to be afraid of the Association, or to refuse it any praise that is its meed. Experience proves it is difficult enough to get a charter for a worthy object, although it may have been supported by the public for years; and a charter to enforce the registration of nurses by a self-constituted association, which has not the support of the managers of all the nurse-training schools, is not likely to be granted by Her Majesty's Privy Council. So long as Westminster, St. Thomas's, Guy's, the London, and the Nightingale Fund, not to speak of Glasgow, Edinburgh, and Dublin, keep as much aloof from the B.N.A. as at present, there is not the slightest hope for the supporters of registration by its agency alone. When Miss Nightingale or any of the true leaders of nursing move in this direction, it will be time enough to give the question serious consideration. A leading superintendent writes that an agent of the B.N.A. "goes worrying nurses till they join, while holding out hopes to them, if they pay their half-crowns annually, they will get situations over their fellows." If so, the end is not far off, as such misleading statements have a habit of coming home to roost. Nurses need not be frightened by any such proceedings, which go to show the emptiness of the larder to which this pushing canvasser offers admission at half-a-crown per head.

### Town Nursing.

A Scheme for Imitation.

The annual meeting of the Manchester and Salford Sick Poor and Private Nursing Institution was held on Monday, December 10th, in the Ardwick Town Hall. In the unavoidable absence of the Mayor of Manchester the chair was taken by the Venerable Archdeacon Anson, president of the committee. The hon. secretary, Dr. Donald, read the report of the General Committee; Miss Potter, hon. secretary of the District Committee, read the report of that branch of the work; and Miss Hertz, hon. secretary of the Ardwick and Ancoats Nurses' Home, that of the latter. Speeches in support of the various resolutions were made by the Venerable Archdeacon Anson, Dr. Tatham, Bishop of Salford (R.C.), Canon Woodhouse, Canon Hicks, and others. The speakers were unanimous in giving the highest praise to the work of the institution, and the way it is carried out, and earnestly advocated its extension by the increase of nurses and the opening of new districts. The fact that the work is one which "helps the poor to help themselves," and thus is the highest kind of charity, was emphasised by several speakers, and Dr. Tatham especially gave his valuable testimony to the effect that the nurses "are of the right kind, and do their work in the right way." In conclusion, a vote of thanks to the officers of the institution was proposed, and carried unanimously.

The Institution is such a good one we cannot forbear giving a sketch of its work. Its objects are: (1) To provide trained nurses for the sick poor in their own homes; (2) to provide trained nurses for those able to pay for their services, any profits arising from this source to be devoted to the former object.

The Institution comprises: (1) District nurses living in homes under trained matrons, and in exceptional cases nurses lodging in their own districts under the supervision of the matron of one of the homes and an honorary lady superintendent; (2) private nurses living in a home under a trained matron.

The order of proceedings for the Private Nurses' Home Committee are as follows: Read, approve, and sign minutes of last meeting; go through nurses' reports from medical men and families; revise the accounts for the month; appoint lady visitor for the month; receive verbal report from the matron; other business.

The order of proceeding for District Committees are: Read, approve, and sign minutes of last meeting; revise the accounts for the month; inspect the time and case books; appoint the lady visitors for the coming months; receive verbal report from the matron; other business.

There is a good service fund for the benefit of nurses who have worked long and well for the institution, and it is given either as increased wages or as a pension.

The Institution comprises three homes: One in Grosvenor Street for private nurses, which produces an annual profit of nearly £100; a home at Salford, where nine nurses live and nurse five districts; and a home at Ardwick, where seven nurses live who are engaged in district work. Five nurses live in lodgings because their districts lie too far away from any of the homes. During the last year the Institution has undertaken five more districts, and has nursed 60 patients for the Manchester Royal Infirmary, 130 cases for St. Mary's Hospital, 200 cases for Salford Hospital, 286 for the Ancoats, and 86 cases for the Children's Hospital, Pendlebury.

Manchester is the only town where the Hospital Sunday Fund gives a grant to the district nurses, yet in spite of this and a legacy of £1,000 from the late Sir Joseph Whitworth, the Sick Poor Institution needs an increase of funds to meet its ever-increasing labour. It is only during the last year that the Salford Home has been opened, and the grouping of nurses together under a lady superintendent instead of leaving them to live in lodgings has answered admirably. They now get through more work, and are able to grapple more complicated cases, and the constant supervision insures more thoroughness throughout. The medical men of the district, to the number of sixteen, sent a testimonial to the committee of the institution, saying that they considered the work of the nurses simply inestimable. In the Ardwick Home the great need is for a nurse to undertake night duty in serious cases, and a special appeal is made for this object. The number of cases nursed during the year by the eight nurses of this home have been 1,144, and the visits paid number 26,737. Of these cases 113 died, and many were sent to convalescent homes, or into the country. No less than 110 of the cases were pneumonia, and we all know that in this disease nursing means life or death. The thoroughness of the rules and working of the Manchester and Salford Sick Poor and Private Nursing Institution, make it a most admirable example of good work well and wisely done, which many towns might copy with advantage.

### Original Articles.

### THE SHUT-IN SOCIETY.

Our neighbours in America are as full of beautiful as of quaint thoughts, and their phraseology is as expressive as it is original. Surely we of the Old World would never have thought of starting a society for invalids with such a queer name as the Shut-in Society, with an advisory board, and a magazine, entitled "The Open Window"? Yet the work of such an association must undoubtedly be a good one, for its objects are to relieve the weariness of the sick-room by sending and receiving letters and other tokens of remembrance; to testify of the love and presence of Christ in the hour of suffering and privation; to stimulate faith, hope, patience, and courage in fellow sufferers, by the study and presentation of Bible promises.

To be a member of this society it is only necessary to be a sufferer shut in from the outside world. There are no fees, no promises required from members; they simply send their names in, and are silently received into the society of their fellow invalids. Every month they receive the small magazine which takes its title from a verse in Genesis, "A window shall thou make," and the well-known lines:—

> "The windows of my soul I throw
> Wide open to the sun."

It is very pathetic reading this open window into the sufferings of those whom the Lord hath shut in. There are original articles and selected quotations;—all very short, as though pain never left the authors free to handle the pen for long together. Then come letters of varying tone: practical hints, requests for prayer, words of rejoicing like the following:—"Dear Shut-ins,—As I had a request published in "The Open Window" that God would restore me to health, I come again to say that I have been healed of all my diseases, after suffering for eight years and four months. I have done a great deal of walking since I have been healed; the longest walk I have taken in one day was a little over six miles. I desire everything that hath breath to help me to praise the Lord for ever and ever, for what he has done for me. Any one of the Shut-ins wishing to ask any questions about my healing, I will be pleased to answer." Here are other specimens of letters from "The Open Window":—"Dear Shut-ins—If those of you who have to spend most of your time in bed will have a large quilt folded, so as to be about a yard wide, and then tightly rolled up and placed under your knees, you will find it a great help If you have never tried it, you have no idea how much it will rest you. I lie that way fully half the time, and find it especially nicer when I wish to work or write. As I am wholly confined to my bed and cannot even be bolstered up, I try every way to get the easiest position. I have always to lie on my back, and can rest much better with the quilt under my knees than any other way I have ever tried. Every few hours I have it taken out for an hour or so, but am always glad to get it back again. I shall be very thankful for tracts and leaflets. I can make good use of a a good many. God bless you one and all."—"My dear Friends,—A few days ago I received a card from the Secretary of the Shut-in Society, informing me that I was a member of the Society. My name has been sent by some unknown friend. Since I am a member of the Society, I would like to give you each and all a greeting in "The Open Window." I may wish you all the strength you need to bear your burdens. I shall always be glad to hear from you, though I am seldom able to write much. I have been shut in for seventeen years." After the correspondence column there comes directions for a weekly "Concert of Prayer," and then a page with the solemn heading, "At Rest." This gives the names of those whom death has released from their shut-in condition. It is in no way so sad as obituary notices usually are; in fact, it is cheerful reading compared with the "Notices and Requests." Here is the simple record of one member's departure: "Mrs. Annie Stevens, of Woburn, Mass., was released from protracted suffering August 1. She was ready and awaiting the messenger. Her cheerfulness and sweet patience made the sick room a pleasant place, especially for little children, whom she loved. Her membership in the Shut-in Society was a source of great comfort to her." A list of books added to the library, of new members, of new addresses, of contributions to the funds, etc., concludes the little magazine. There is not a single paragraph in the eighteen pages which is not characterised by singular thoughts and whimsical expressions, which we recognise as essentially American. But under it all there is that absence of reserve, that total lack of affectation and shyness which always astonishes us Old World fogies. Where is the English man or woman who would throw open the window of their soul? The more stricken an Englishman, the more bitter his sufferings, the further does he withdraw into his inner self: he seeks no sympathy, no open window, he chooses to shut himself in even more closely than suffering necessitates. How far we miss our mission of consolation to our fellow beings through this national reserve is a debateable point; certainly some of us who are acquainted with sickness, either as sufferers ourselves, or as attendants on the ill and dying, might be none the worse for a little of the frank taking and giving of consolation which marks the members of the Shut-in Society. And now in return for the many beautiful thoughts culled from "The Open Window," we are going to send to our neighbours over the seas a quotation from a source from which they do not seem to have drawn: "My friends, wait upon God. When He himself shall give the signal and release you from this service, then are ye released unto Him. But for the present bear to dwell in this place, wherein He has set you. Short, indeed, is this time of your sojourn, and easy to bear for those that are so minded."—*Epictetus. Diss.* i., IX.

### Presentation.

Miss Reynolds, for many years lady superintendent of Adelaide Hospital, Dublin, was, on the occasion of her resignation last week, presented by the nursing staff with a gold watch and chain, and by the medical staff with a dressing bag and surgical case.

### Notice.

On Saturday, December 29th, at 4.45 p.m., a children's Christmas Service will be held in the chapel of the Hospital for Sick Children, Great Ormond-street, Bloomsbury.

### Wages and Washing.—IV.

(*Concluded from page* xliii.)

### Points for Consideration by Committees and Nurses.

The question of a nurse's worth and wages then, resolves itself to this: Nurses are worse paid than domestic servants. If a girl wishes to enter domestic service she can begin early in her teens to earn from £10 to £20 a-year, and by the time she is twenty, she will probably be earning £25 a-year. A nurse cannot begin her training as a probationer till she is twenty-five, and her wages, at that age, usually commence at £10. Then the domestic servant can continue in service till she is seventy if she is well preserved, whereas hospital nursing tries a woman of fifty, and even private nursing cannot be carried on after sixty. Here, then, is the fact that if a girl in search of a vocation puts wages first, she had better learn to cook or scrub; her duties will be easier and her pay will be better; for who ever expects a domestic servant to pay her own washing, or live on meat once a day? From a purely economic standpoint, it is evident there is something wrong when skilled labour cannot command a higher wage than unskilled labour; and the difficulty is not to recognise the wrong, but to discover the reason of it, and to provide the remedy.

Why are nurses so poorly paid? Because they can just supply their immediate wants on their scanty wages, and they never look to the future, or take up the cudgels on their own behalf. There is no profession among men so poorly paid as is nursing among women. What roars of laughter would greet the writer who would dare to compare the fortunes of the valet or footman with those of the physician or surgeon! Of course the idea is utterly ridiculous, yet not much more so than that of comparing the wages of the housemaid with those of the hospital nurse. A woman wants a means of livelihood, and has a natural desire to seek that work which will call forth her highest and best powers. This is all very well for the woman with a private income, or the woman who hopes some day to marry; but those who have to depend on their own earnings had better hesitate ere entering a hospital. For the few years they are there they will secure sufficient for the day, but there is for most of them a future—and not a pleasant one. If it is a wonder where all the medical students go, is it not also a wonder where all the worn out nurses go? The matrons of large hospitals, and the promoters of the Pension Fund, could give a few startling answers to that last question. The reason, then, why nurses' wages are so small is because they do not look to the future, and are too easily content.

And what is the remedy? Well, to persuade nurses to combine for their own good, to remember that the time comes when a woman's strength fails her, and she can work no more. If only nurses would remember this, they would petition committees without remorse, until free unlimited washing was given them, and thus a small sum was secured to invest by in the Pension Fund for old age. Let it be thoroughly understood that it is not charity a nurse seeks, but merely adequate wages for her work, which is surely worthy of better pay. No work could be harder, more wearing for both mind and body; the public is only too fond of pointing to nurses as the highest and best of their sex, and of lavishing on them praises and presents. But what a nurse wants is merely the acknowledgment that as a labourer she is worthy of her hire, and that she ought by right to be able to provide for old age from the earnings of her own hands. We have given statistics on this question of Wages and Washing, and we have given our own deductions from these statistics. We shall now be glad to receive and print the opinions of our readers who have considered the subject.

### Everybody's Opinion.

*Correspondence on all subjects is invited. No communications can be entertained if the name and address of the correspondent is not given, or unless one side of the paper only be written on.*]

### AN APPEAL FOR CHRISTMAS HELP

Mrs. Allison, Lady Superintendent of Hull Royal Infirmary, writes: As Christmas is now approaching, I am anxious to make known to the many friends of the infirmary that I propose having the annual Christmas tree in the Children's Ward as usual. I shall be very glad to receive and acknowledge gifts of toys and any other articles suitable for distribution amongst the adult as well as juvenile patients, or money to buy the same. It is only by a generous response to a public appeal that enough gifts can be procured for about one hundred and fifty poor sick people, who are perforce away from their homes during the festive season. Trusting that you may be able to find space for this letter in your valuable columns.

### TRAINED OR NOT?

An Outsider writes: The above question in respect to the Chichester Infirmary is causing widespread comment. The local papers are all taking it up with great zeal, and I am glad to state that even the "wise editor" of the "Chichester Observer" devoted a very excellent leader to the question in last week's paper. The feeling in the City of Chichester and neighbourhood is very strong. Great is the praise bestowed upon Mr. Bostock for his courage in bringing to the front this weighty question, in spite of the stumblingblocks put in his way. I can with true honesty say probationers cannot be trained at the Chichester Infirmary. It is no use beating about the bush. Under the present management a probationer cannot receive a medical or surgical training. It is quite impossible, for the place is throughout in a deplorable state—barring the financial question. "T. A.'s" remarks in your last issue are perfectly correct: it is not for efficiency or necessary qualifications many matrons are appointed. Not long ago a woman was appointed matron of a seaside hospital, on condition that she first went to a London hospital for three months' training. The staff nurses at Chichester are styled "sisters," yet the one on the female side has not yet finished her three years' training. It is strange that the Chichester Infirmary has been allowed to go on as it has done for so long, and it is a good thing for it and all its inmates that at last its faults have been made public, for now we may hope to see a speedy remedy.

Note.—"T. A." requests us to state that her remarks on "Nurses and Presents" reached us before "Handclasp's" letter appeared in print, and that "T. A." is therefore quite innocent of the charges implied against her.—We must remind our readers who persist in writing to us on this subject that we cannot re-open the discussion.

### Appointments.

[It is requested that successful candidates will send a copy of their applications and testimonials, with date of election, to The Editor, The Lodge, Porchester Square, W.]

Bucks County Infirmary.—A sub-committee of this infirmary went carefully through all the applications for the post of matron, and on December 5th Miss Purchase was elected by a majority of votes.

Hospital for Sick Children.—Miss Annesley Kenealy has been appointed sister of the Helena and Alexandra wards of the Hospital for Sick Children, Great Ormond Street. Miss Kenealy trained at St. Bartholomew's, and from there went to the Orthopædic Hospital aud Nervous Infirmary, Philadelphia. Next Miss Kenealy went to the Child's Hospital, New York; so that she brings to her work in London a thorough knowledge of the American hospital system. Miss Kenealy's testimonials are excellent.

### Notes and Queries.

### Queries.

(88) *Health Lectures.*—Are the "Edinburgh Health Lectures" published, and if so, by whom, at what price?—Sister Victoria.

(89) *Health Lectures.*—Are the "Coombe Lectures" published, and if so, by whom, at what price?—Sister Victoria.

(90) *Somerv lle Club.*—Would any nurse who belongs to the Somerville Club for Ladies kindly state if she finds it useful and interesting?—Middlesex.

(91) *Who Trains Male Nurses?*—Mr. F. J. Guildford writes: Can you inform me of an institution or hospital for training male nurses? Your doing so at your convenience will greatly oblige.

(92) *Home Wanted for Boy 15, Healthy, but Slightly Imbecile.*—I am anxious to find a home for a boy 15 years of age, who is slightly imbecile, but has perfect health, hearing, sight, &c. He is the son of very poor parents, but a relative has offered to pay £20 a-year to any institution that would give him the peculiar kind of teacning that he requires.

### Jottings.

How to Stand.—Mr. Ellis writes from 6, Clarence Street, Gloucester, to request us to correct a statement that his article on "How to Stand" in *Nursing Notes* was "reproduced without acknowledgment" in another paper which deals with nursing. It was to a considerable extent re-written, and a diagram added, specially for the use of the paper in question.

An Explanation.—Our attention has been called to the fact that some statements made in the review of *Nursing Notes* in our last issue are not accurate. We have ascertained the facts to be that in the paper referred to the substance of an article, "A Few Words on Sponges," which appeared in *Nursing Notes* of July 1st, was subsequently reproduced, its original source being mentioned. Again, an article by Mr. T. S. Ellis, whose explanation is given above, was printed in the same paper on another occasion, with an intimation from Mr. Ellis that his views had been expressed elsewhere, it having previously appeared almost verbatim in *Nursing Notes*. It will be apparent, therefore, that the statement made in our notice that the articles referred to were reproduced "entire," and "without acknowledgment," is not correct. Finding this to be the case, we offer our sincere apologies to Mr. Ellis and to anyone else who may justly consider themselves aggrieved by the unintentional publication of the inaccuracies in question.

Telegraphic Addresses.—The telegraphic address of the private nursing staff of Fitzroy House, under Mrs. Bluett's control, is "Anstiseptic, London." This will be useful information to many of our readers. Most nursing institutions now have a telegraphic address, and we shall be glad to publish a list for the guidance of those members of the profession and the public who employ nurses, if the authorities will send us the necessary particulars.

The Value of "The Hospital."—A lady superintendent writes: "I have had no difficulty whatever in selecting twelve good nurses (certificated and trained) from our most numerous applicants. The advertisement in The Hospital brought us by far the most replies.' This is very satisfactory, and proves that the employment of uncertificated and imperfectly trained nurses by nursing institutions is not only undesirable, but wholly unnecessary, besides being an injustice to the large number of deserving nurses who have spent time and money in perfecting themselves for the work. Chichester Infirmary authorities please take the hint!

### Vacancies.

The following vacancies are announced:—

### Matrons.

Llanelly Hospital; £30.

Bolton Infirmary and Dispensary; £75,

Jersey Dispensary and Infirmary; £25.

### Sister.

Borough Fever Hospital, Leeds; £30.

### District Nurses.

Ryde, I.W.

Wrexham, N. Wales.

Gloucester.

### Nurses.

Nursing Institution, 96, 97, 98, Wimpole Street, London, W., Mr. Wilson has several vacancies for thoroughly-trained Nurses.

Plumstead Infirmary; £20.

Brompton Consumption Hospital; two; £25.

Hartlepool Hospital (night); £25.

Southern Hospital, Manchester; £25.

Southport Infirmary; £20.

Kent Nursing Institution; several.

Stephen Monckton Nurses' Home; several.

Home Hospital, Eastbourne.

Royal Hospital for Children and Womem, Waterloo Bridge Road; several.

Southport Trained Nurses' Home; several.

Nurses' Home, Beckenham.

Cheltenham Nursing Institution.

Swansea Nursing Institution.

Windsor Royal Infirmary; £20.

Grimsby Hospital Home.

Nurses' Home, Newcastle-on-Tyne; several.

City Infectious Hospital, Newcastle-on-Tyne; several; £25.

Hampstead Home Hospital; two; £23.

Nursing Home, Bishop's Stortford; £20.

Guildford County Hospital; £12.

Cottage Hospital, Eltham.

Christ's Hospital, Hertford; £20.

Rhyl Hydro., North Wales.

Home for Epileptics, Manor House, Maghull, near Liverpool.

### Midwives.

Rivers Street Home, Bath.

Newlands, Ryde.

Manchester Maternity Hospital; two.

### Probationers.

Guildford County Hospital.

Royal Hospital for Children and Women, Waterloo Bridge Road; several.

Princess Alice Hospital, Eastbourne.

Kent Nursing Institution.

Hampstead Home Hospital.

